# Recombinant BCG Overexpressing *phoP-phoR* Confers Enhanced Protection against Tuberculosis

**DOI:** 10.1016/j.ymthe.2018.08.023

**Published:** 2018-09-01

**Authors:** Sang Kyun Ahn, Vanessa Tran, Andrea Leung, Mark Ng, Ming Li, Jun Liu

**Affiliations:** 1 Department of Molecular Genetics, Faculty of Medicine, University of Toronto, Toronto, ON M5G 1M1, Canada

**Keywords:** BCG, tuberculosis, phoP-phoR

## Abstract

The live tuberculosis vaccine *Mycobacterium bovis* BCG (Bacille Calmette-Guérin) comprises a number of genetically distinct substrains. In BCG-Prague, *phoP* of the PhoP-PhoR two-component system is a pseudogene due to a single insertion mutation. We hypothesized that this mutation partially accounts for the low immunogenicity of BCG-Prague observed in the 1970s. In this study, we showed that complementation with the *M. bovis* allele of *phoP* restored BCG-Prague’s immunogenicity. Furthermore, we showed that overexpression of the *M. bovis* allele of *phoP-phoR* in BCG-Japan, a strain already containing a copy of *phoP-phoR*, further enhanced immunogenicity and protective efficacy. Vaccination of C57BL/6 mice with the recombinant strain rBCG-Japan/PhoPR induced higher levels of interferon-γ (IFN-γ) production by CD4^+^ T cells than that with the parental BCG. Guinea pigs vaccinated with rBCG-Japan/PhoPR were better protected against challenge with *Mycobacterium tuberculosis* than those immunized with the parental BCG, showing significantly longer survival time, reduced bacterial burdens, and less severe pathology. Taken together, our study has identified a genetic modification that could be generally applied to generate new recombinant BCG vaccines.

## Teaser

Tuberculosis (TB) is the leading infectious disease. BCG (Bacille Calmette-Guérin), a live vaccine that has been used for nearly 100 years, has a limited efficacy against TB. Liu and colleagues now show that the protective efficacy of BCG can be significantly improved by overexpressing the two-component signal transduction system *phoP-phoR* in BCG.

## Introduction

Despite global health efforts, tuberculosis (TB), caused by *Mycobacterium tuberculosis* (Mtb), remains a major cause of mortality worldwide. The lack of a protective vaccine, the emergence of drug-resistant Mtb strains, and the high rate of Mtb/HIV coinfection continue to fuel the TB epidemic. Bacille Calmette-Guérin (BCG) is the only licensed TB vaccine. Although it is effective against disseminated forms of TB in children,^1^, ^2^ BCG has limited protection against pulmonary TB in adults, the most common and contagious form of the disease. Clinical studies have shown variable efficacies ranging from 0% to 80%.^3^, ^4^, ^5^

One hypothesis to explain the variable efficacy of BCG concerns the heterogeneity of the BCG strains.^6^ BCG was derived from a virulent strain of *Mycobacterium bovis* (*M. bovis*) through *in vitro* passaging from 1908 to 1921. Subsequent worldwide distribution and continuous passaging until the 1960s resulted in a number of BCG substrains. Genetic differences among BCG strains including deletions and duplications of genomic regions and SNPs have been well documented.^7^, ^8^, ^9^, ^10^, ^11^, ^12^ Whether these differences affect effectiveness of BCG against TB is a matter of debate,^6^, ^13^ and no clear evidence is currently available to recommend the use of one particular strain over the others due to the paucity of clinical trials directly comparing multiple BCG strains.^14^

A number of studies in humans have demonstrated strain-dependent variations in immune response induced by BCG.^14^ Although the cell-mediated immunity required for protection against TB is not fully understood, it involves multiple components including CD4^+^ and CD8^+^ T cells.^15^, ^16^, ^17^ BCG induces a T helper cell 1 (Th1) type response, mostly interferon-γ (IFN-γ) production by CD4^+^ T cells.^18^ Traditionally, immunogenicity of BCG was determined by measuring the tuberculin (purified protein derivatives [PPDs] of Mtb) sensitivity induced by the vaccine in children who were tuberculin-negative before vaccination.^19^ Although its use as a surrogate measure of protection has been questioned in recent years,^20^, ^21^ tuberculin reactivity continues to be used as an *in vivo* assay to evaluate the cell-mediated immune responses and as a marker for immunogenicity.^22^, ^23^ Supporting this, a strong association between tuberculin reactivity and PPD-specific IFN-γ levels has been reported in BCG-vaccinated infants.^24^ Furthermore, tuberculin reactivity and IFN-γ production were found to be non-redundant and complementary measures of anti-TB immunity in young people.^25^

Interestingly, studies in the 1970s found that BCG-Prague consistently exhibited significantly lower tuberculin reactivity than the other 10 BCG strains tested in children and guinea pigs.^26^, ^27^ Due to the concern over its low immunogenicity, BCG-Prague was replaced by BCG-Russia in Czechoslovakia in 1981, after nearly 30 years of use.^19^ Factors causing this reduced tuberculin reactivity are unknown. However, we found that *phoP* in BCG-Prague is a pseudogene, containing a 1-bp insertion that disrupts the C-terminal DNA-binding domain.^9^ This mutation is specific to BCG-Prague and is not detected in other BCG strains.^9^, ^28^ PhoP is a response regulator of the PhoP-PhoR two-component system, and it positively regulates more than 40 genes in Mtb, including two T cell antigens (Ag85A, PPE18) that have been used to construct subunit vaccines.^17^, ^29^ As such, we hypothesize that the low immunogenicity of BCG-Prague could be a result of the *phoP* mutation.^9^ In this study, we show that complementation with the *M. bovis* allele of *phoP* indeed restored BCG-Prague’s immunogenicity. More importantly, we also demonstrate that overexpression of the *M. bovis* allele of *phoP-phoR* in BCG-Japan, already possessing a copy of *phoP-phoR*, further increased immunogenicity and protective efficacy of this vaccine strain. Taken together, our studies suggest that overexpression of *phoP-phoR* could be a generally applicable approach to improve effectiveness of the BCG strains.

## Results

### Overexpression of *phoP* or *phoP-phoR* in BCG Strains Increases Immunogenicity

**Figure 1:**
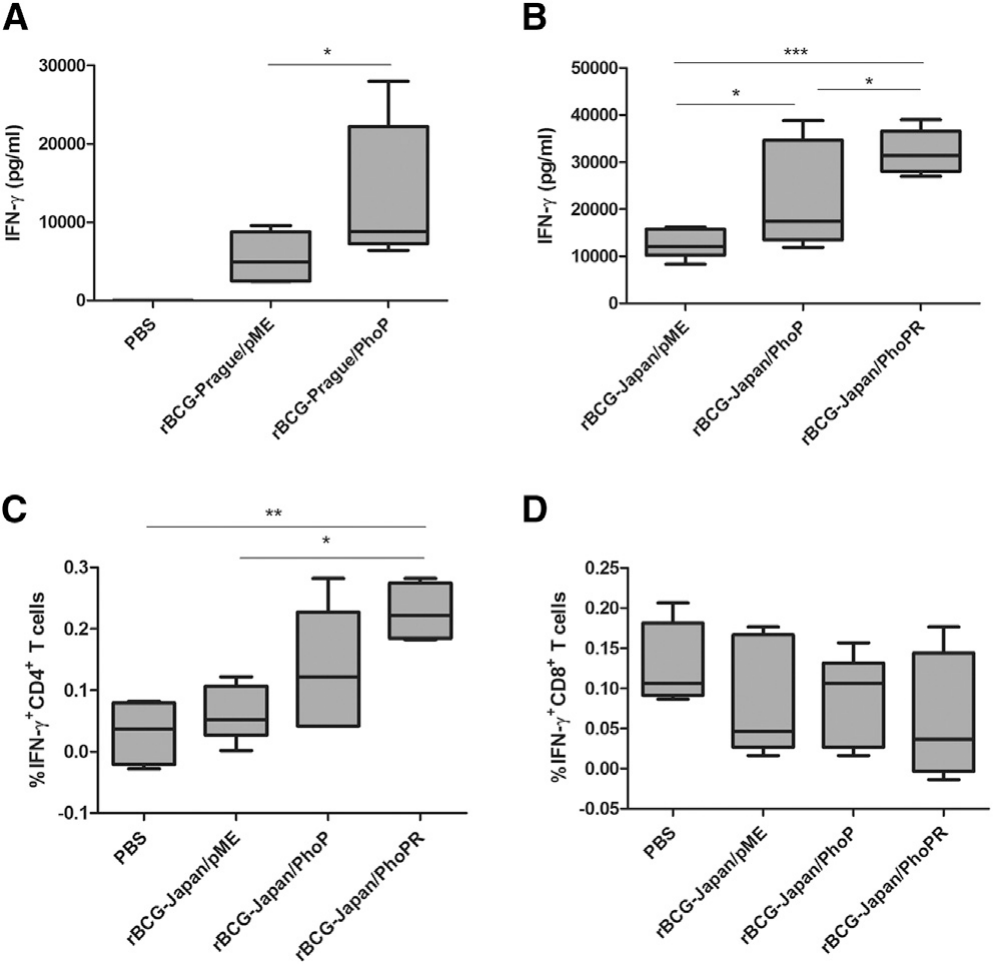
Overexpression of *phoP* or *phoP-phoR* in BCG Induces IFN-γ Production (A and B) C57BL/6 mice were immunized subcutaneously with 5 × 10^4^ CFUs of the recombinant BCG Prague strains (A) or the recombinant BCG-Japan strains (B). At 8 weeks post-vaccination, splenocytes were harvested and incubated with or without PPD (10 μg/mL) for 72 hr, and cytokine production was analyzed by ELISA. Data are plotted as box-whiskers in which the whiskers represent the minimum and maximum of all data (n = 4 mice). (C and D) C57BL/6 mice were immunized with the indicated rBCG-Japan strains. Harvested splenocytes were stimulated with or without PPD (25 μg/mL) for 24 hr and were then stained for T cell surface markers CD3-PE, CD4-FITC (C) or CD8a-PercyPCy5.5 (D) and intracellular IFN-γ (IFN-γ-APC), followed by FACS analysis. Data are plotted as box-whiskers (n = 4 mice), after subtraction of reading from samples without PPD stimulation. In (A), the two-tailed unpaired Student’s t test was performed. In (B) and (C), one-way ANOVA and Bonferroni multiple comparison tests were performed. *p < 0.05; **p < 0.01; ***p < 0.001.

To determine the effect of the *phoP* mutation on BCG immunogenicity, we first complemented BCG-Prague with the *M. bovis* allele of *phoP*. The *M. bovis* allele of *phoP-phoR* is identical to that of BCG-Pasteur (Figure S1). Therefore, we used the genomic DNA of BCG-Pasteur as the template for PCR amplification and cloning. The *phoP* gene was cloned into a multicopy shuttle vector pME and introduced into BCG-Prague (rBCG-Prague/PhoP). C57BL/6 mice were vaccinated with the recombinant BCG-Prague strain, and the production of PPD-specific IFN-γ was measured by ELISA. The rBCG-Prague/PhoP strain induced a higher level of PPD-specific IFN-γ release in C57BL/6 mice, which was ∼2.4-fold of that in the mice immunized with the parental strain (p < 0.05; Figure 1A).

To test whether overexpression of *phoP* in a BCG strain already containing a copy of *phoP* could further improve immunogenicity, we chose BCG-Japan as a parental strain to construct recombinant BCG. BCG-Japan is one of the most widely used BCG vaccines in the world and has a superior safety record in clinical studies partially because of the loss of lipid virulence factors phthiocerol dimycocerosates (PDIMs) and phenolic glycolipids (PGLs).^9^, ^30^, ^31^, ^32^ BCG-Japan is also considered to be more immunogenic because it contains fewer genetic deletions than other BCG strains.^8^, ^33^ BCG-Japan contains *phoP*-*phoR*, and PhoR is a histidine kinase that activates PhoP by phosphorylation upon stimulation by low pH.^29^, ^34^ Therefore, in addition to constructing a recombinant BCG-Japan strain overexpressing *phoP* alone (rBCG-Japan/PhoP), we also constructed a strain overexpressing both *phoP* and *phoR* to maintain the functional ratio of this two-component system. The *M. bovis* allele of *phoP-phoR* was cloned in pME and transformed into BCG-Japan to generate rBCG-Japan/PhoPR.

Consistent with the results obtained with BCG-Prague, both rBCG-Japan/PhoP and rBCG-Japan/PhoPR induced significantly higher levels of PPD-specific IFN-γ production in C57BL/6 mice, which were 1.7- and 2.6-fold, respectively, of that in the mice immunized with the parental strain (Figure 1B). Among the three strains, rBCG-Japan/PhoPR appeared to induce the highest level of IFN-γ.

Of note, BCG-Prague exhibited reduced IFN-γ induction (rBCG-Prague/pME, 5,430 ± 1,132 pg/mL) compared with BCG-Japan (rBCG-Japan/pME, 12,537 ± 1,236 pg/mL), but overexpression of *phoP* in BCG-Prague elevated the IFN-γ production (rBCG-Prague/PhoP, 12,855 ± 3,143 pg/mL) to the level comparable with that of BCG-Japan (Figures 1A and 1B). These results support our hypothesis that the *phoP* null mutation is at least partially responsible for the lower immunogenicity BCG-Prague exhibited compared with other BCG strains in previous studies.^26^, ^27^ For further studies, we decided to focus on the recombinant BCG-Japan strains because BCG-Japan was observed to be naturally more immunogenic than BCG-Prague, and the recombinant BCG-Japan strains displayed even more promising immunogenicity.

To determine the source of IFN-γ induced by the recombinant BCG-Japan strains, we performed intracellular cytokine staining and fluorescence-activated cell sorting (FACS) analyses. We found that CD4^+^ T cells were likely responsible for the enhanced IFN-γ release (Figure 1C). The frequency of IFN-γ-producing CD4^+^ T cells in the mice vaccinated with rBCG-Japan/PhoPR was ∼3.5-fold of that in the mice vaccinated with the parental strain, agreeing with the fold difference in total IFN-γ production (∼2.6 fold) between these two groups (Figures 1B and 1C). The rBCG-Japan/PhoPR strain also appeared to induce more IFN-γ-producing CD4^+^ T cells than rBCG-Japan/PhoP, but the difference was not statistically significant.

Neither of the BCG strains induced a robust CD8^+^ T cell response compared with the sham-immunization control (Figure 1D). No significant induction of other cytokines (interleukin [IL]-2, tumor necrosis factor [TNF], IL-12, IL-4, IL-5, and IL-10) by the recombinant BCG-Japan strains was detected (Figure S2).

Taken together, these results suggest that the *phoP* mutation is partially responsible for the low immunogenicity of BCG-Prague. More importantly, overexpression of the *M. bovis* allele of *phoP* or *phoP-phoR* in a BCG strain containing a chromosomal copy of *phoP-phoR* (e.g., BCG-Japan) further boosted the ability of the vaccine to induce antigen-specific IFN-γ production by CD4^+^ T cells, suggesting that this is a generally applicable approach to improve BCG immunogenicity.

### rBCG-Japan/PhoPR Is Safe in SCID mice

**Figure 2:**
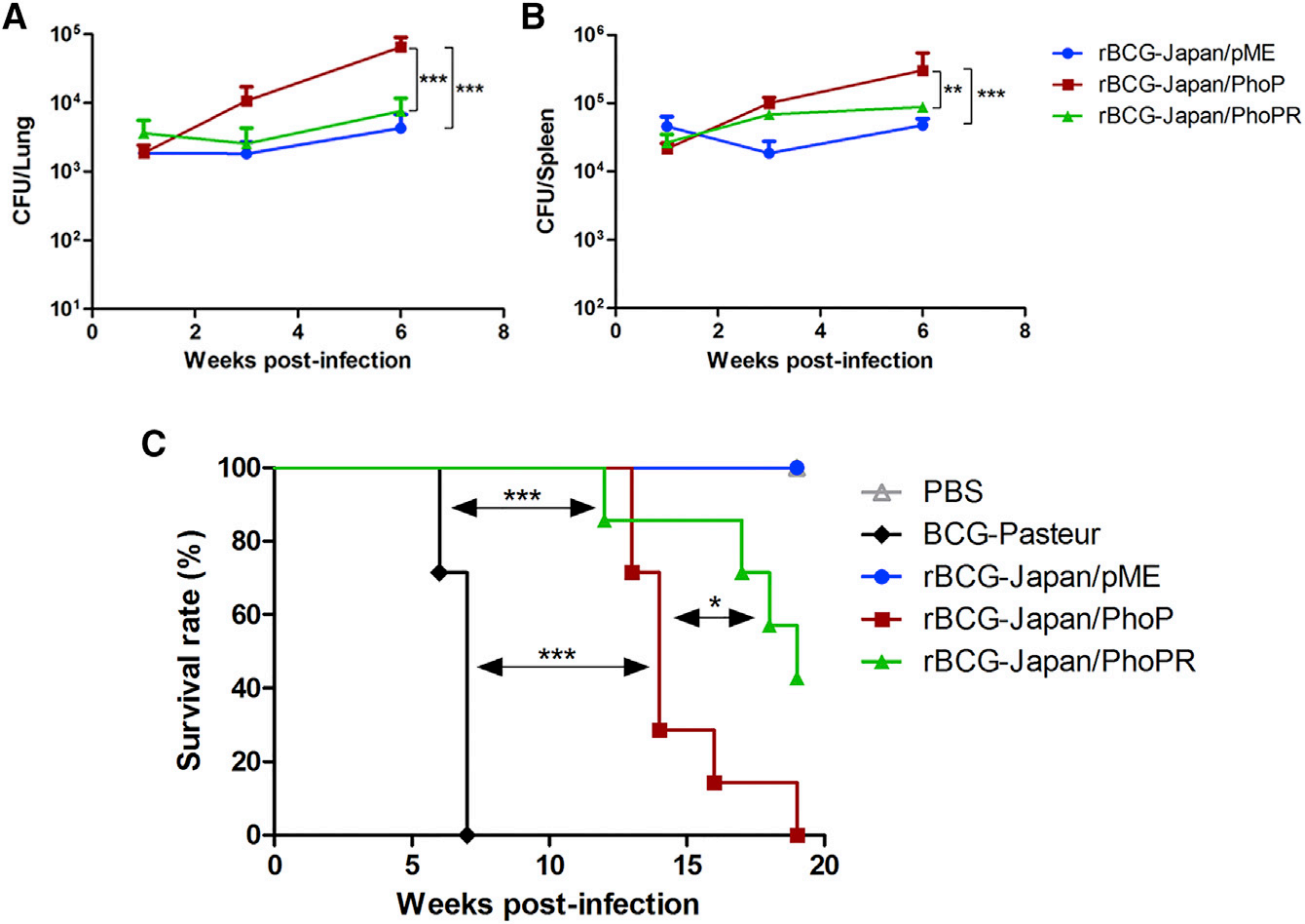
Safety Profile of rBCG-Japan/PhoPR in SCID Mice (A and B) SCID mice (n = 16 per group) were infected intravenously with 10^5^ CFUs of the recombinant BCG-Japan strains (rBCG-Japan/PhoP and rBCG-Japan/PhoPR) or rBCG-Japan/pME as the control. At day 1 post-infection, four mice from each group were sacrificed to assess the initial infection doses, which were 2,677 ± 1,028, 2,080 ± 889, and 2,765 ± 965 CFUs/lung (mean ± SD) for rBCG-Japan/pME, rBCG-Japan/PhoP, and rBCG-Japan/PhoPR, respectively. At weeks 1, 3, and 6 post-infection, mice (n = 4 per group) were euthanized and bacterial burdens in the lungs (A) and spleen (B) were determined. Data are shown as mean ± SD (n = 4 SCID mice). Two-way ANOVA and Bonferroni multiple comparison tests were performed. (C) SCID mice (n = 10 per group) were infected intravenously with 10^7^ CFUs of the indicated BCG strains. Three mice from each group were euthanized at day 1 post-infection to assess infection doses, which were 19,833 ± 14,530, 25,558 ± 10,335, 22,125 ± 7,730, and 28,200 ± 14,418 CFUs/lung (mean ± SD) for BCG-Pasteur, rBCG-Japan/pME, rBCG-Japan/PhoP, and rBCG-Japan/PhoPR, respectively. The remaining animals (n = 7 per group) were monitored weekly until they reached a humane endpoint. Survival curves were plotted using the Kaplan-Meier method. Log rank test was performed to compare each pair of the survival curves. *p < 0.05; **p < 0.01; ***p < 0.001.

Because PhoP is a known virulence factor of Mtb,^35^ overexpression of the *M. bovis* allele of *phoP* or *phoP-phoR* in BCG-Japan may increase virulence and compromise safety. To address this, we first infected severe combined immunodeficiency (SCID) mice with the recombinant BCG-Japan strains and monitored bacterial growth in target organs for up to 6 weeks. Interestingly, there was no significant difference between the growth of rBCG-Japan/PhoPR and the parental BCG in the lungs or spleen over the course of the experiment (Figures 2A and 2B). On the other hand, overexpression of *phoP* alone increased replication of BCG-Japan in SCID mice compared with both the parental strain and rBCG-Japan/PhoPR, suggesting that overexpression of *phoP* may increase virulence of BCG-Japan.

To further evaluate the safety of the recombinant BCG-Japan strains, we performed a long-term SCID mice survival experiment. BCG-Pasteur was also included in this experiment for comparison. The median survival times of SCID mice infected with BCG-Pasteur, rBCG-Japan/PhoP, and rBCG-Japan/PhoPR were 7, 14, and 19 weeks, respectively (Figure 2C). All SCID mice infected with the parental strain or PBS survived until week 20, when the experiment was terminated. Log rank analysis revealed that the rBCG-Japan/PhoP group had significantly reduced survival compared with the rBCG-Japan/PhoPR group (p = 0.02) and the parental group (p < 0.001), which is consistent with the higher bacterial burdens observed in this group in the short-term infection experiment (Figures 2A and 2B). The rBCG-Japan/PhoPR group also showed reduced survival compared with the parental group (p = 0.02). Importantly, both rBCG-Japan/PhoPR and rBCG-Japan/PhoP were significantly less virulent than BCG-Pasteur (p < 0.001) in SCID mice. Taken together, these results suggest that the overexpression of the *M. bovis* allele of *phoP-phoR* does not increase replication of BCG-Japan in SCID mice and causes only a modest increase in virulence. Consistently, rBCG-Japan/PhoPR was cleared from the immunocompetent C57BL/6 mice at the same rate as the parental strain (Figure S3).

### rBCG-Japan/PhoPR Confers Superior Protection over Parental BCG

To examine whether overexpression of *phoP*-*phoR* or *phoP* alone improves BCG-mediated protection against Mtb infection, we first conducted a short-term (8 weeks) guinea pig infection experiment. Groups of six guinea pigs were vaccinated with the recombinant BCG-Japan strains (rBCG-Japan/PhoP or PhoPR), the parental BCG (rBCG-Japan/pME), or sham-immunized with PBS. This was followed by aerosol infection with a high dose (5,000 colony-forming units [CFUs]/lung) of Mtb H37Rv. Four guinea pigs reached the humane endpoint (loss of 15% maximal body weight and/or labored breathing), including two in the PBS group at week 5 post-infection, one in the parental BCG group at week 6, and one in the rBCG-Japan/PhoP group at week 7. In contrast, none of the guinea pigs vaccinated with rBCG-Japan/PhoPR reached the humane endpoint for the duration of this experiment.

**Figure 3:**
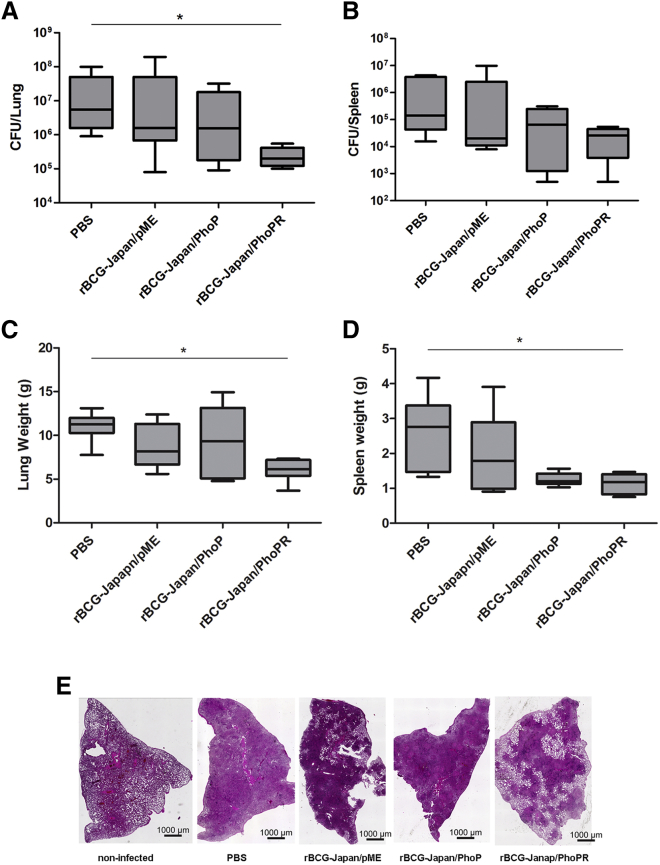
rBCG-Japan/PhoPR Reduces Mtb Burden in Guinea Pigs Guinea pigs were vaccinated with recombinant BCG-Japan strains and aerogenically challenged with Mtb H37Rv (∼5,000 CFUs/lung). The infection dosage was predetermined by guinea pig infection experiments. The same batch and dilution of Mtb H37Rv cultures were used to infect the experimental groups under the predetermined parameter setting of GlasCol nebulizer. The Mtb burdens (A and B), organ weights (C and D), and lung pathology (E) were determined after 8 weeks of infection. In (A)–(D), data are plotted as box-whiskers (n = 6 guinea pigs), and one-way ANOVA and Bonferroni multiple comparison tests were performed. *p < 0.05.

Guinea pigs were euthanized once they reached the humane or experimental endpoint (week 8 post-infection), and their lungs and spleen were obtained for further analysis. The guinea pigs vaccinated with rBCG-Japan/PhoPR had on average ∼2.0 log_10_ lower Mtb counts in their lungs compared with those in the PBS group (p < 0.05) or the parental BCG group (Figure 3A). The difference between the rBCG-Japan/PhoPR and parental BCG groups was approaching statistical significance (p = 0.065, two-tailed unpaired Student’s t test). No significant difference was found between the rBCG-Japan/PhoP and PBS groups, or between the rBCG-Japan/PhoP and the parental BCG groups. Similarly, the guinea pigs in the rBCG-Japan/PhoPR group generally had reduced Mtb burden in their spleen compared with those in the other groups, although the differences were not statistically significant (Figure 3B).

In addition to increased Mtb burdens, increased organ weights (lungs and spleen) have been associated with more severe disease phenotypes and frequently observed in guinea pigs infected with virulent Mtb.^36^, ^37^ Among the four groups, the rBCG-Japan/PhoPR group had the lowest lung and spleen weights, and the difference between the rBCG-Japan/PhoPR and the PBS groups was statistically significant (p < 0.05; Figures 3C and 3D).

Severe tissue damage highlighted by extensive infiltration and visible granulomatous lesions was observed in the lungs of the guinea pigs that were unvaccinated or vaccinated with either the parental or rBCG-Japan/PhoP strains (Figure 3E). Only partial infiltration and lesions in the lungs were observed from the guinea pigs in the rBCG-Japan/PhoPR group, suggesting less tissue damage occurred in these animals.

To further evaluate the protective efficacy of the recombinant BCG-Japan strains, we performed a long-term (10 months) guinea pig survival experiment. We decided to focus on rBCG-Japan/PhoPR because rBCG-Japan/PhoP had a more concerning level of virulence while not displaying superior protection compared with rBCG-Japan/PhoPR in the aforementioned experiments. Guinea pigs (12 per group) were vaccinated with rBCG-Japan/PhoPR, the parental strain, or PBS and were challenged aerogenically with ∼1,000 CFUs/lung of Mtb H37Rv 8 weeks post-vaccination. Guinea pigs were euthanized at the humane endpoint or an experimental endpoint (43 weeks post-infection), and survival curves were plotted using Kaplan-Meier analysis.

**Figure 4:**
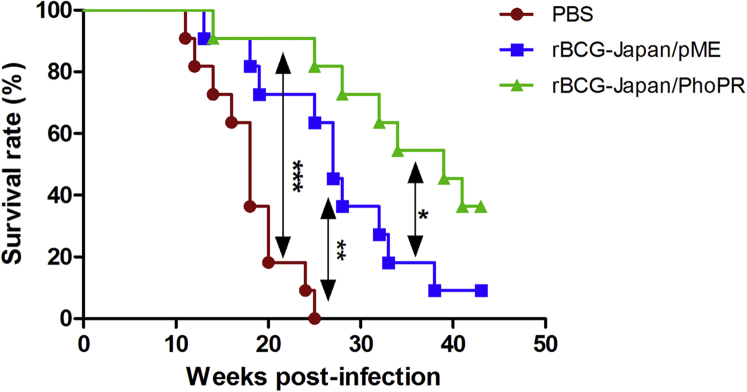
rBCG-Japan/PhoPR Prolongs the Survival of Guinea Pigs Infected with Mtb Guinea pigs (n = 12 animals per group) were vaccinated with rBCG-Japan/PhoPR, the parental strain, or PBS and aerogenically challenged with Mtb H37Rv (∼1,000 CFU/lung). Four guinea pigs were randomly selected and sacrificed at day 1 post-infection to determine the actual infection dosage, which was 840 ± 184 CFUs/lung (mean ± SD). The remaining animals (n = 11 per group) were monitored weekly until they reached a humane endpoint or an experimental endpoint (43 weeks post-infection). Survival curves were plotted using the Kaplan-Meier method. Log rank test was performed to compare each pair of the survival curves. *p < 0.05; **p < 0.01; ***p < 0.0001.

The median survival times for the PBS, parental, and rBCG-Japan/PhoPR groups were 18, 27, and 39 weeks, respectively (Figure 4). Log rank analysis revealed that the rBCG-Japan/PhoPR group survived significantly longer than the parental BCG group (p < 0.05) and the PBS group (p < 0.0001). The parental BCG group also survived significantly longer than the PBS group (p < 0.01).

Compared with sham immunization, the parental BCG vaccine prolonged the survival of guinea pigs by 9 weeks, whereas rBCG-Japan/PhoPR prolonged the survival of guinea pigs by 21 weeks, which is a 133% improvement over the parental BCG. At week 43 post-infection, when the experiment was terminated, only one guinea pig in the parental BCG group survived compared with four in the rBCG-Japan/PhoPR group. All animals in the PBS group succumbed to infection by week 25.

The guinea pig lungs and spleen were further analyzed after the animals were euthanized at the humane or experimental endpoint. As expected, there was no significant difference on bacterial burdens in animals from different groups that were sacrificed at the humane endpoint, with ∼10^6^ CFUs Mtb in both lung and spleens. The Mtb counts in the five guinea pigs (four in the rBCG-Japan/PhoPR group and one in the parental BCG group) euthanized at the experimental endpoint were ∼2 log_10_ lower in both lungs and spleen.

**Figure 5:**
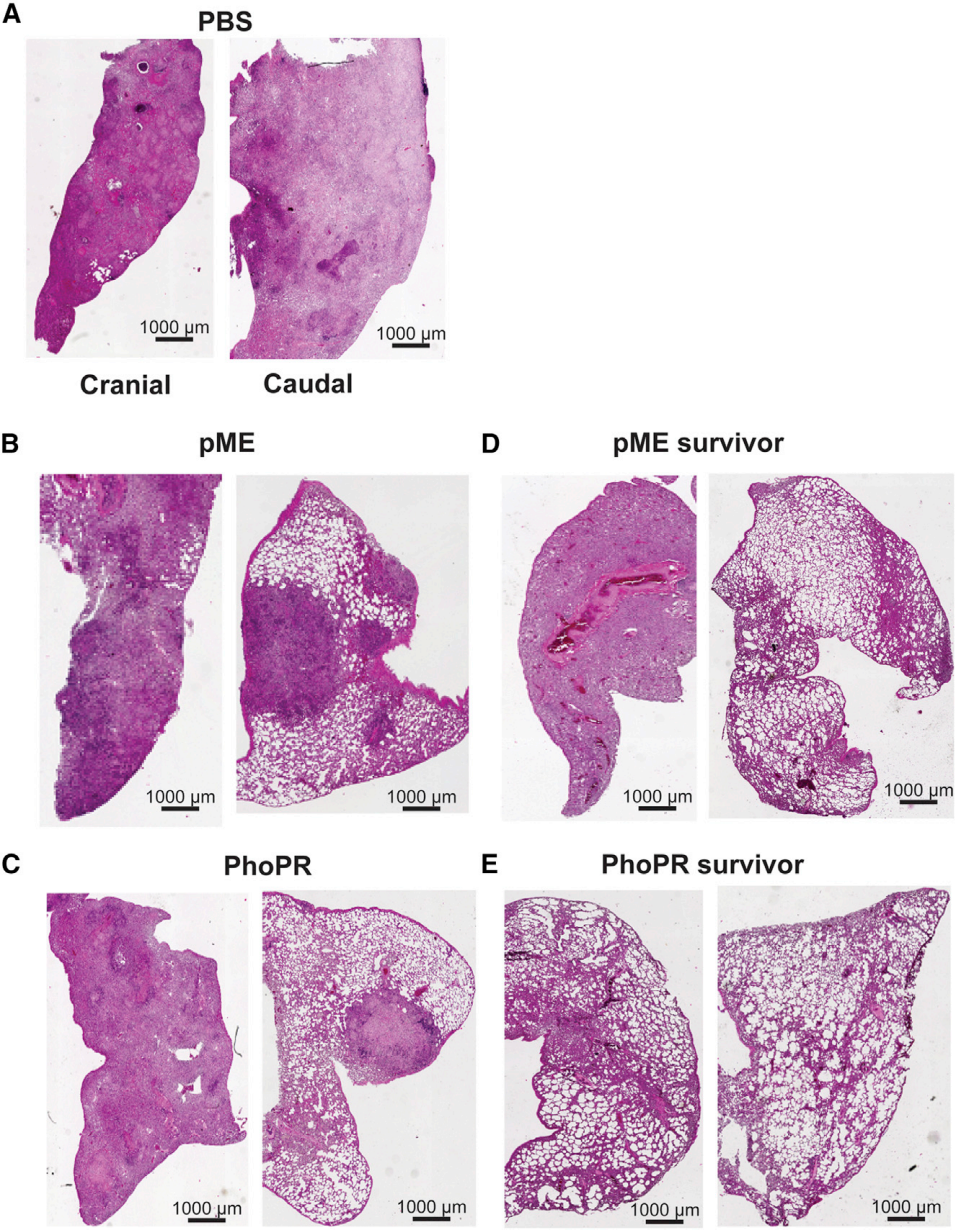
rBCG-Japan/PhoPR Reduces the Lung Pathology of Guinea Pigs Infected with Mtb Five guinea pigs from the long-term survival experiment were chosen for histological analysis. These include one guinea pig from the PBS group (A), the pME group (B), and the PhoPR group (C) that reached the humane endpoint at weeks 25, 27, and 39, respectively. The pME (B) and PhoPR (C) guinea pigs represent the sixth animal to be euthanized from each group (median). The sole survivor from the pME group (D) and one randomly chosen survivor from the PhoPR group (E) were also included. For each guinea pig, sections of caudal and cranial lobes of the left lungs were analyzed by H&E staining.

The lungs of five guinea pigs were subjected to histological analysis. They included one animal from each group that reached the humane endpoint, the sole survivor from the parental BCG group, and one of the four survivors from the rBCG-Japan/PhoPR group. Interestingly, the mortality of guinea pigs appeared to be associated with the extent of tissue damage in the caudal lobe. The three animals euthanized before the experimental endpoint had extensive (Figure 5A) or partial consolidation (Figures 5B and 5C) in the caudal lobes in addition to extensive consolidation in the cranial lobes. In contrast, the two survivors appeared to have healthy tissues in the caudal lobe, despite the fact that the one from the parental BCG group also had severe tissue damage in the cranial lobe (Figure 5D). Strikingly, one of the randomly selected survivors from the rBCG-Japan/PhoPR group appeared to have normal lungs with no visible consolidation in either lobe (Figure 5E).

Taken together, these data demonstrated that rBCG-Japan/PhoPR confers superior protection over the parental BCG strain.

### Transcriptional Analysis of rBCG-Japan/PhoPR and rBCG-Japan/PhoP

Previously, the PhoP regulon has been identified by comparing transcriptomes of the Δ*phoP* and wild-type (WT) strains of Mtb by DNA microarray or RNA sequencing (RNA-seq).^29^, ^38^ To determine the effect of overexpression of the *M. bovis* allele of *phoP-phoR* or *phoP* on the BCG-Japan transcriptome, we conducted RNA-seq and compared gene expression of exponentially growing rBCG-Japan/PhoPR and rBCG-Japan/PhoP with that of the parental strain.

Compared with the parental strain, there were 102 upregulated (Table S1, worksheet 1) and 468 downregulated (Table S1, worksheet 2) genes (≥1.5-fold, *Q* value < 0.05) in rBCG-Japan/PhoP. This observation is consistent with the previous studies in which a dual-regulatory role of PhoP has been demonstrated in Mtb.^29^, ^38^ Importantly, statistically significant overlap was observed between our RNA-seq data and previous transcriptome analyses.^29^, ^38^ For example, microarray analysis by Walters et al.^29^ showed that 44 genes were upregulated in WT Mtb compared with Δ*phoP*, and 28 of them overlapped with the genes upregulated in rBCG-Japan/PhoP (p = 1.3E−35). Similarly, 68 genes were induced by the presence of PhoP in WT Mtb according to the RNA-seq data by Solans et al.,^38^ and 33 of them overlapped with the genes upregulated in rBCG-Japan/PhoP (p = 1.2E−37). It is noteworthy that a total of 27 genes were common in all three datasets (Figure S4A).

A smaller number of genes were differentially expressed in rBCG-Japan/PhoPR because this strain had 31 upregulated genes (Table S1, worksheet 3) and 229 downregulated genes (Table S1, worksheet 4) compared with the parental strain. Again, there are significant overlaps between the genes upregulated in rBCG-Japan/PhoPR and the PhoP-activated genes in Mtb identified by previous studies,^29^, ^38^ with 16 genes common in all three datasets (Figure S4B). Moreover, expression patterns of the PhoP regulated genes in rBCG-Japan/PhoPR also demonstrated good consistency with the previous literature (Table S1, worksheets 5 and 6).

A substantially larger number of genes were downregulated in rBCG-Japan/PhoP (468 genes) or rBCG-Japan/PhoPR (229 genes) compared with the number of genes negatively regulated by PhoP in Mtb identified by Walters et al.^29^ (70 genes) and Solans et al.^38^ (72 genes). This discrepancy could be partially due to the fact that the previous studies compared the Mtb strains that either possess (WT) or completely lack (Mtb Δ*phoP*) functional PhoP, whereas our experiments examined the effects of the *phoP* or *phoP-phoR* overexpression in a BCG strain already containing a chromosomal copy of these genes. Because PhoP controls the expression of other regulatory elements such as members of the *whiB* family transcription factors (*whiB3*, *whiB5*, *whiB6*), global regulator *lsr2*, and non-coding RNA *mcr7*,^29^, ^34^, ^38^ it is possible that many of these altered gene expressions are an indirect result of the *phoP* or *phoP-phoR* overexpression. In addition, sequence polymorphisms have been found between the *M. bovis* and Mtb alleles of *phoP-phoR* (Figure S1),^39^ which could contribute to the observed difference.

## Discussion

Strategies to improve TB vaccines include the development of subunit and live-attenuated vaccines, and there are currently 16 vaccine candidates being tested under clinical trials.^17^, ^40^ Most of these candidates are subunit vaccines for which selected Mtb antigens are expressed in replication-deficient viral vectors or are administered as purified protein/adjuvant combinations. A large number of Mtb antigens have been tested, but none of them have proved to be superior to BCG in animal models.^41^, ^42^, ^43^, ^44^, ^45^, ^46^ As a result, subunit vaccines are currently evaluated more as a booster rather than a replacement of BCG.^17^, ^40^ MVA85A has recently completed a phase IIb trial as the first subunit candidate to reach efficacy testing.^47^ Unfortunately, the results were rather disappointing because MVA85A was unsuccessful in providing significantly improved protection against TB or Mtb infection to BCG-vaccinated South African infants. This failure has raised questions regarding plausibility of the subunit vaccine approach and further emphasized the importance of whole-cell, live-vaccine research.^48^

A number of approaches have been explored to develop new live vaccines including various recombinant BCGs and attenuated Mtb strains. Of these, only a few have proven to be superior to current BCG in the animal models, including the three vaccines (rBCG30, VPM1002, and MTBVAC) that have entered clinical trial evaluation as a possible BCG replacement.^17^, ^22^, ^35^, ^49^, ^50^ The rBCG30 vaccine is a recombinant BCG-Tice strain that overexpresses antigen Ag85B. When challenged by Mtb, the guinea pigs vaccinated with rBCG30 exhibited reduced bacterial burden (by 0.5–1.0 log_10_) and prolonged survival compared with those immunized with the parental BCG strain.^22^, ^51^ However, this effect was specific to BCG-Tice because overexpression of Ag85B in BCG-Connaught did not result in improved protection.^51^ Although rBCG30 completed a phase I trial in 2004,^52^ no further development has been reported. VPM1002 is a recombinant BCG strain that expresses listeriolysin of *Listeria monocytogenes*, and it entered phase IIa trials in 2014.^17^ The rationale behind this vaccine was the notion that listeriolysin could facilitate phagosomal escape of BCG into the cytosol of macrophages, thereby increasing antigen presentation.^49^ The BALB/c mice vaccinated with VPM1002 showed a reduction in Mtb burden by 0.5–1.0 log_10_ compared with those immunized with the parental strain.^49^, ^53^ However, improved protection was not observed with the guinea pig model because VPM1002 did not prolong the survival of the Mtb-challenged animals compared with the parental strain.^54^ A *phoP* deletion mutant of Mtb was also evaluated as a vaccine candidate with the reasoning that attenuated Mtb may share more antigens with clinical strains of Mtb than BCG.^35^ Compared with BCG, Mtb Δ*phoP* provided similar protection in mice but better protection in guinea pigs against Mtb challenge.^35^, ^55^ To ensure safety of the vaccine, *fadD26* was additionally deleted to further attenuate the strain and generate MTBVAC, which showed a comparable safety profile to BCG-Pasteur or BCG-Danish in SCID mice.^56^ MTBVAC recently completed the phase Ia trial and was shown to have similar safety to BCG in healthy adults.^57^

In this study, we adopted a different approach to construct live TB vaccines based on previous clinical and genomic studies. We hypothesized that genetic differences among BCG strains could contribute to the differential immune response induced by BCG, and that identification of these genetic factors might lead to novel approaches to develop new and improved TB vaccines. Although more than a dozen studies have compared the immune response induced by different BCG strains in humans, only two or three BCG strains were examined at a time in the majority of these studies.^14^, ^58^ When comparing only a few BCG strains, a large number of genome polymorphisms among these strains make it highly difficult to pinpoint the genetic mutation responsible for the antigenic profile. Moreover, a number of key experimental differences such as the choice of BCG strains, patients’ age at immunization, and population size have led to inconclusive results.^14^ Nonetheless, a study led by the WHO in the 1970s compared 11 BCG strains in children and found that BCG-Prague was an outlier in terms of its lower tuberculin reactivity compared with the other tested BCG strains, including BCG-Danish, -Pasteur, -Glaxo, -Japan, -Russia, and -Moreau.^26^ Investigation of the genome sequences of these BCG strains allowed us to identify the pseudogenization of *phoP* in BCG-Prague,^9^, ^28^, ^33^ and it led us to hypothesize that this mutation contributes to BCG-Prague’s reduced immunogenicity. Consistent with our hypothesis, we showed that complementation of BCG-Prague with the *M. bovis* allele of *phoP* restored immunogenicity. We further demonstrated that overexpression of the *M. bovis* allele of *phoP-phoR* in a BCG strain already containing *phoP-phoR* (BCG-Japan) increased IFN-γ production by CD4^+^ T cells and improved protection against the Mtb challenge. Taken together, our data suggest that overexpression of *phoP-phoR* can be a generally applicable method to improve the protective efficacy of BCG. This approach, possibly in combination with other genetic modifications, can be highly beneficial in developing new recombinant BCG vaccines against TB. Future studies to apply this method to other BCG strains (e.g., BCG-Pasteur, BCG-Russia) will provide additional validation. Because overexpression of *phoP-phoR* in BCG-Japan modestly increases its virulence, the potential benefit and risk need to be carefully evaluated when choosing specific BCG strains to construct recombinant BCG overexpressing *phoP-phoR*.

Besides the *phoP* mutation in BCG-Prague, mutations in *phoR* have been identified in several other BCG strains compared with *M. bovis*, including an 11-bp deletion in BCG-Sweden and -Birkhaug, a 10-bp deletion in BCG-Danish and -Glaxo, and a 1-bp deletion in BCG-Frappier.^9^, ^28^ On the other hand, the sequence of *phoR* in BCG-Pasteur, -Phipps, -Tice, -Prague, -Japan, or -Russia is identical to that of *M. bovis*. In several studies that compared the cytokine and T cell profiles in infants vaccinated with BCG-Japan or -Danish, it was shown that distinct immune responses were induced by these two strains.^58^, ^59^, ^60^, ^61^ It will be interesting to determine whether the *phoR* mutation in BCG-Danish contributes to this observation. In addition, a recent study found that the PhoP-PhoR system of *M. bovis* is impaired in its function compared with the one in Mtb due to SNPs in this locus (Figure S1).^39^ Therefore, it will be of great interest to examine whether overexpression of the Mtb allele of *phoP-phoR* in BCG could result in even greater protection.

One of the major challenges in TB vaccine development is the lack of an immunological correlate of protection or “biomarker” for efficacy.^62^, ^63^, ^64^ Multiple studies have shown that IFN-γ is required for immunity against Mtb,^65^, ^66^, ^67^, ^68^ and accordingly, identification of the Mtb antigens that induce strong IFN-γ production has been a main strategy to select candidates for constructing subunit vaccines.^41^, ^42^, ^43^, ^44^, ^45^, ^46^ However, a number of studies have found no correlation between BCG-induced IFN-γ production and protection,^62^, ^69^ and the lack of boosting effect by MVA85A in BCG-vaccinated infants also adds uncertainty to the role of IFN-γ as a biomarker.^47^ Nonetheless, in our study, the enhanced protection of rBCG-Japan/PhoPR appears to be associated with an increased production of IFN-γ by CD4^+^ T cells. There are two possible explanations for our observations. First, it is possible that the level of IFN-γ induced by the current BCG strains is too low and needs to reach a certain threshold in order to have a linear correlation with protection. Among the BCG strains that were compared in infants, BCG-Japan appears to induce Th1 cytokines (IFN-γ, TNF, IL-2) and CD4^+^ T cells better than BCG-Danish and -Russia.^58^, ^61^ A retrospective analysis of cohorts in Kazakhstan found that BCG-Japan was also more effective in reducing the risk for TB in infants than BCG-Russia or BCG-Serbia.^70^ Consistently, in our study, rBCG-Japan/PhoPR induced ∼2- to 3-fold higher levels of IFN-γ than the parental strain, thereby resulting in better protection. Second, the enhanced IFN-γ production induced by rBCG-Japan/PhoPR was detected in splenocytes stimulated by PPD (which is a protein mixture), rather than by a single antigen (e.g., Ag85A). Immune response induced by a broad range of antigens such as PPD is more favorable because it is more likely to mimic natural Mtb infection.

Currently, the exact mechanisms by which overexpression of *phoP-phoR* in BCG-Japan improves the immunogenicity remain unknown. A number of genes that were induced in rBCG-Japan/PhoP and rBCG-Japan/PhoPR encode proteins involved in lipid metabolism. For example, the operons composed of *pks2-papA1-mmpL8* (*Rv3825c*-*Rv3823c*) and *pks3-pks4-papA3-mmpL10* (*Rv1180*-*Rv1183*) are involved in biosynthesis and translocation of sulfolipids (SLs), 2,3-diacyltrehaloses (DATs), and penta-acyltrehaloses (PATs).^71^, ^72^ These lipids play a structural role in the cell envelope, and they were shown to promote the ability of Mtb to persist in the infected host and modulate host immune responses.^73^ However, these glycolipids were reported to be absent from the avirulent strain of Mtb H37Ra and from BCG.^74^, ^75^ Consistently, we did not detect SLs, DATs, and PATs in BCG-Pasteur, BCG-Japan, and rBCG-Japan strains using a solvent system specifically for these glycolipids in two-dimensional thin-layer chromatography (2D-TLC) analyses.^76^ Previously we showed that BCG-Japan does not produce PDIMs and PGLs,^30^ which are caused by a mutation in *ppsA*.^77^ Overexpression of *phoP* or *phoP-phoR* in BCG-Japan did not result in the production of PDIMs or PGLs (Figures S5A and S5B). There was no difference in the content of phosphatidylinositol mannosides (PIMs) among the rBCG-Japan strains (Figure S5C). Taken together, these data suggest that overexpression of *phoP* or *phoP-phoR* in BCG-Japan does not alter the cell wall lipid composition. On the other hand, enzymes involved in lipid metabolism have been shown to be potent T cell antigens, such as the Ag85 family antigens (Ag85A, Ag85B, and Ag85C), which are mycolyl transferases and have been included in various forms of vaccine construction. It is possible that enzymes involved in the biosynthesis and translocation of SLs, DATs, and PATs (e.g., Pks2-4, PapA1 and PapA3, MmpL8, and L10) are also T cell antigens, and that increased expression of these enzymes observed in rBCG-Japan/PhoPR may collectively contribute to its increased immunogenicity and protective efficacy.

Guinea pigs are the gold standard animal model for testing TB vaccine efficacy during pre-clinical development.^54^, ^78^, ^79^ The pathogenesis of disease, pathological lesions, and response to BCG vaccination are similar to those described in humans. Promisingly, the superior protection of rBCG-Japan/PhoPR was demonstrated in this model. It was clearly evident that the guinea pigs immunized with this vaccine showed significantly prolonged survival and reduced pathology, which are hallmarks for testing novel vaccines in animal models. In addition, the safety of rBCG-Japan/PhoPR was confirmed in SCID mice, which are highly immunocompromised and are the reference model for evaluating live vaccines.^80^, ^81^ There is consensus that novel TB vaccine candidates must meet criteria to advance from the discovery stage into pre-clinical development. These criteria include a robust induction of IFN-γ, better protective efficacy than current BCG, and a comparable safety profile to the BCG in the established animal models.^81^ We propose that the rBCG-Japan/PhoPR strain described in this study satisfies these criteria and thus is a promising candidate for future clinical development as a BCG replacement. Future studies to combine rBCG-Japan/PhoPR with subunit vaccines as its booster will also determine its full potential.

## Materials and Methods

### Bacterial Strains and Culture Conditions

*M. bovis* BCG and Mtb H37Rv were grown at 37°C in Middlebrook 7H9 broth supplemented with 0.2% glycerol, 10% albumin-dextrose-catalase, and 0.05% Tween 80 or on 7H11 agar supplemented with 0.5% glycerol and 10% oleic acid-albumin-dextrose-catalase. Plasmid manipulation and propagation were performed using *Escherichia coli* DH5α grown in Luria-Bertani broth or agar. Kanamycin was added at a concentration of 50 μg/mL for *E. coli* or 25 μg/mL for BCG.

### Construction of Expression Vectors for *phoP* and *phoP*-*phoR*

BCG-Pasteur allele of *phoP-phoR* was cloned into the expression vector. The *phoP* gene was amplified using BCG-Pasteur genomic DNA as a template. The 1,028-bp product containing *phoP*, as well as the 257-bp upstream region of the *phoP* translational start site, was obtained using a forward primer phoP-F (5′-AAAAAGGTACCGCTTGTTTGGCCATGTCAAC-3′) and a reverse primer phoP-R (5′-AAAAACTGCAGGCTGCCGATCCGATTAACTAC-3′), which contain a KpnI and a PstI restriction site (underlined), respectively. Using these restriction sites, the PCR product was ligated to a shuttle vector pME (pME-PhoP).

Similarly, a vector that expresses the *phoP*-*phoR* operon (pME-PhoPR) was constructed by cloning a 2,501-bp PCR product, containing *phoP* and *phoR* (as well as the intergenic region between the two genes, 177 bp upstream of the *phoP* start codon and 78 bp downstream of *phoR* stop codon), into pME. This product was amplified from BCG-Pasteur genomic DNA using forward primer phoPR-F (5′-AAAAAGGTACCGGTCGCAATACCCACGAG-3′) and reverse primer phoPR-R (5′-AAAAACTGCAGCCTCAGTGATTTCGGCTTTG-3′) containing a KpnI and a PstI site (underlined), respectively. The constructs were confirmed by DNA sequencing and were electroporated into BCG.

### Ethics Statement

All of the animal procedures were approved by the University of Toronto Animal Care Committee (Animal Use Protocols 20011640 and 20011379). All experimental procedures were performed in accordance with the Canadian Council on Animal Care (CCAC) and University of Toronto regulations.

### Analysis of Immunogenicity in C57BL/6 Mice

Female C57BL/6 mice were purchased from Charles River Laboratories and were age-matched (6 weeks) within each experiment. Four mice per group were inoculated subcutaneously on the scruff of the neck with approximately 5 × 10^4^ CFUs of BCG strains in 0.2 mL of PBS/0.01% Tween 80. Control mice were given 0.2 mL of PBS/0.01% Tween 80. After 8 weeks, mice were euthanized to isolate splenocytes and measure intracellular IFN-γ. In brief, splenocytes were seeded at 2 × 10^6^ cells/well in 100 μL in triplicate and stimulated with 2.5 μg/well of PPD (Statens Serum Institute, Denmark) or complete RPMI as a control and incubated at 37°C and 5% CO_2_. After 19 hr of stimulation, GolgiPlug (BD Biosciences, Canada) was added in a 1:1,000 final dilution and incubated for an additional 5 hr. After a total of 24 hr stimulation, plates were centrifuged at 1,400 rpm for 5 min at 4°C. The supernatant was removed and the cell pellet was washed in 200 μL of FACS buffer (0.5% BSA/PBS), resuspended in Fc Block (eBioscience, Canada) diluted in FACS buffer (1:400), and incubated for 15 min on ice in the dark. An additional 150 μL of FACS buffer was added and mixed, and plates were then centrifuged at 1,400 rpm for 5 min at 4°C. Supernatant was removed and cells were stained for extracellular T cell surface markers (CD3-phycoerythrin [PE], CD4-fluorescein isothiocyanate (FITC), and CD8a-PercyPCy5.5 from BD Biosciences, Canada) diluted in FACS buffer and incubated for 30 min on ice in the dark. Following the extracellular marker staining, the cells were washed with 150 μL of FACS buffer and permeabilized and fixed with 1× CytoFix/CytoPerm (BD Biosciences, Canada) for 20 min. Cells were then washed with 1× PermWash (BD Biosciences, Canada) and incubated with IFN-γ-allophycocyanin (APC) (BD Biosciences, Canada) for 30 min to stain for intracellular IFN-γ. Cells were centrifuged as above, resuspended in 200 μL FACS buffer, and analyzed on a BD FACSCalibur flow cytometer (BD Biosciences, Canada). A total of 300,000 events per sample were collected in the lymphocyte gate and analyzed using FlowJo V7.6. Gates for analysis were set based on isotype controls.

Quantitative measurements of IFN-γ production were also determined using an ELISA using the OptEIA Mouse IFN-γ ELISA set (BD Biosciences, Canada). Samples used for ELISAs were supernatants from splenocytes stimulated by PPD (10 μg/mL) for 72 hr.

### Protection against Mtb Challenge in Guinea Pigs

#### Short-Term Bacterial Burden Assay

Groups of six female outbred Hartley guinea pigs (200–250 g) were purchased from Charles River Laboratories, and they were vaccinated subcutaneously with 5 × 10^4^ CFUs of BCG-Japan containing pME (rBCG-Japan/pME), pME-PhoP (rBCG-Japan/PhoP), or pME-PhoPR (rBCG-Japan/PhoPR) in 0.2 mL PBS/0.01% Tween 80 or PBS/0.01% Tween 80 alone as a control. At 8 weeks post-vaccination, guinea pigs were challenged with 5,000 CFUs/lung of Mtb H37Rv by an aerosol route using a GlasCol nebulizer. The infection dosage was predetermined by guinea pig infection experiments. The same batch and dilution of Mtb H37Rv cultures were used to infect the experimental groups under the predetermined parameter setting of GlasCol nebulizer. At 8 weeks post-challenge, guinea pigs were euthanized to obtain the lungs and spleen. A portion of the spleen and the cranial lobe of the left lung was fixed in 10% formalin for histological analysis. The remaining portion of the lungs and spleen was homogenized separately and plated on 7H11 agar to quantify the Mtb burden in each organ. Colonies were counted after incubation at 37°C for 3 weeks.

#### Long-Term Survival Assay

Groups of 12 female Hartley guinea pigs were vaccinated as described above, and they were aerogenically infected with ∼1,000 CFUs of Mtb H37Rv at 8 weeks post-vaccination. The infection dosage (∼1,000 CFUs/lung) was predetermined by guinea pig infection experiments. The same batch and dilution of Mtb H37Rv cultures were used to infect the experimental groups under the predetermined parameter setting of GlasCol nebulizer. In addition, four guinea pigs were randomly selected and sacrificed at day 1 post-infection to determine the actual infection dosage, which was 840 ± 184 CFUs/lung (mean ± SD). The remaining animals (n = 11 per group) were monitored weekly until they reached a humane endpoint (loss of 15% maximal body weight and/or labored breathing) or an experimental endpoint (43 weeks post-infection). The guinea pigs that reached the endpoints were euthanized, and their lungs and spleen were harvested. The spleen and the entire right lung were homogenized separately and plated for Mtb quantification as described above. A portion of the spleen and cranial and caudal lobes of the left lung was fixed in 10% formalin for histological analysis.

### Histological Analysis

Formalin-fixed tissues were embedded into paraffin blocks at the Centre of Modeling Human Disease (Toronto Centre for Phenogenomics). Serial sections (5 μm thick) were prepared and they went through the deparaffinization process with three changes of xylene (3 min each) before being rehydrated with four washes of alcohol (100%, 100%, 95%, 70%, 3 min each). Sections were stained with H&E (EMD Chemicals, Canada) and examined using Cytation 5 (BioTek, Canada).

### Analysis of BCG Virulence in SCID Mice

#### Short-Term Bacterial Burden Assay

Female Fox Chase CB17 SCID mice (Charles River Laboratories) were age-matched (7 weeks old) in this experiment. Groups of 16 mice were infected intravenously via a lateral tail vein with 10^5^ CFUs of rBCG-Japan/pME, rBCG-Japan/PhoP, or rBCG-Japan/PhoPR in 0.2 mL of PBS/0.01% Tween 80 or PBS/0.01% Tween 80 alone as a control. At day 1 (to assess the initial infection doses), as well as weeks 1, 3, and 6 post-infection, the lungs and spleen were harvested from the euthanized mice (four mice at each time point), homogenized in PBS, and plated on 7H11 agar to observe bacterial burden in each organ. Colonies were counted after incubation at 37°C for 3–4 weeks.

#### Long-Term Survival Assay

Groups of 10 age-matched female SCID mice (7 weeks old) from Charles River Laboratories were infected intravenously via a lateral tail vein with 10^7^ CFUs of BCG-Pasteur, three aforementioned recombinant BCG-Japan strains, or PBS/0.01% Tween 80 as described above. Three mice from each group were euthanized at day 1 post-infection (for assessing infection doses), whereas the remaining mice (n = 7 per group) were monitored weekly until they reached a humane endpoint (loss of 20% maximal body weight).

### C57BL/6 Clearance Assay

Groups of four male C57BL/6 mice (Charles River Laboratories) were age-matched (6 weeks) and injected intravenously with 10^8^ CFUs of rBCG-Japan/pME or rBCG-Japan/PhoPR in 0.2 mL of PBS/0.01% Tween 80 or PBS/0.01% Tween 80 alone as a control. At days 1, 14, 21, and 42 post-infection, mice were euthanized. The harvested organs were homogenized separately and plated on 7H11 agar to assess bacterial burden.

### RNA Extraction and Illumine Sequencing

Recombinant BCG-Japan strains (rBCG-Japan/PhoP, rBCG-Japan/PhoPR) and the parental strain (rBCG-Japan/pME) were grown in 50 mL of 7H9 media containing 25 μg/mL kanamycin at 37°C to OD_600_ ∼1.5. The bacterial cultures were pelleted and washed three times with PBS. Total RNA (1–3 μg) was isolated using the RNeasy Mini Kit (QIAGEN, Canada) and purified using the RNAClean XP Kit (Beckman Coulter, Canada) and RNase-Free DNase Set (QIAGEN, Canada) according to the manufacturer’s instructions. Purified RNA was used to construct the cDNA library according to the TruSeq Stranded RNA LT Guide from Illumina. The concentration and size distribution of the cDNA library were analyzed by Agilent 2100 Bioanalyzer, and the average library size was approximately 350 bp. High-throughput sequencing was carried out on an Illumina HiSeq 2500 system according to the manufacturer’s instructions (Illumina HiSeq 2500 User Guide), and 150-bp paired-end reads were obtained. The raw reads were filtered by Seqtk and then mapped to BCG-Japan reference sequence (ftp://ftp.ncbi.nlm.nih.gov/genomes/all/GCF/001/287/425/GCF_001287425.1_bcg_Tokyo/) using Bowtie2 (version: 2-2.0.5).^82^ Counting of reads per gene was performed using HTSeq followed by TMM (trimmed mean of M values) normalization.^83^, ^84^ Differentially expressed genes were defined as those with a false discovery rate (*Q* value) <0.05 and fold-change >1.5 using the edgeR software.^85^

### Lipid Analysis by TLC

Cell wall lipids were examined using 2D-TLC, according to published procedures.^30^, ^76^, ^86^ In brief, the apolar and polar lipids were extracted from BCG cells (50 mg of dry mass) and analyzed on silica gel 60 plates (EMD Chemicals). For detection of PDIMs, apolar lipids were developed with the solvent system A: petroleum ether/ethyl acetate (98:2, v/v, 3×) in the first dimension and petroleum ether/acetone (98:2) in the second dimension. Lipids were visualized by staining plates with 5% phosphomolybdic acid followed by gentle charring. For detection of PGLs, the apolar lipid extract was developed with the solvent system C: chloroform/methanol (96:4) in the first dimension and toluene/acetone (80:20) in the second dimension, followed by charring with α-naphthol. For detection of PIMs, polar lipids were separated with the solvent system E: chloroform/methanol/water (60:30:6) in the first dimension and chloroform/acetic acid/methanol/water (40:25:3:6) in the second dimension, followed by charring with α-naphthol. For detection of SLs, DATs, and PATs, the polar and apolar lipid extracts were separated with the solvent system D: chloroform/methanol/water (100:14:0.8) in the first dimension and chloroform/acetone/methanol/water (50:60:2.5:3) in the second dimension, followed by charring with α-naphthol and 5% phosphomolybdic acid, respectively.

## Author Contributions

J.L. conceptualized the study. S.K.A., V.T., A.L., and J.L. designed experiments. S.K.A., V.T., A.L., M.N., and M.L. performed experiments, and S.K.A., V.T., A.L., and J.L. analyzed the data. S.K.A. and J.L. wrote the original draft, and S.K.A., V.T., M.N., and J.L. reviewed and edited the manuscript. J.L. secured funding and provided supervision.

## Conflicts of Interest

The authors declare no competing financial interests.

## Supplemental Information

Document S1. Figures S1–S5

Table S1. Differential Gene Expression Analysis between the Parental Strain (rBCG-Japan/pME) and rBCG-Japan/PhoP or rBCG-Japan/PhoPR

Document S2. Article plus Supplemental Information
