# Supplementary material for: Recombinant BCG Overexpressing phoP-phoR Confers Enhanced Protection against Tuberculosis
Source: Mol Ther. 2018 Sep 1;26(12):2863–74. doi: 10.1016/j.ymthe.2018.08.023 (PMC6277425; doi:10.1016/j.ymthe.2018.08.023)
Supplement: Document S1. Figures S1–S5 [file mmc1.pdf]

YMTHE, Volume *26*

Supplemental Information

Recombinant BCG Overexpressing *phoP-phoR* Confers Enhanced Protection against Tuberculosis

Sang Kyun Ahn, Vanessa Tran, Andrea Leung, Mark Ng, Ming Li, and Jun Liu

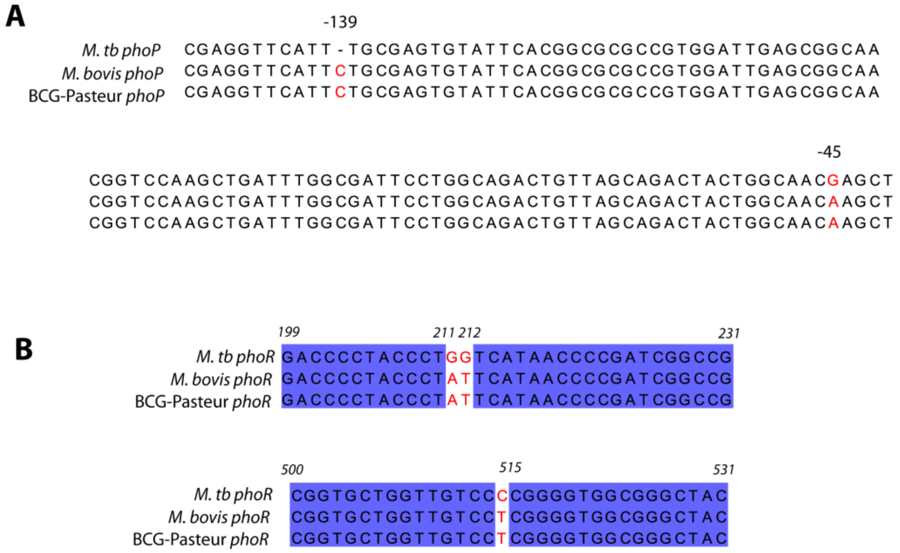

Figure S1. Sequence alignment of the *phoP-phoR* loci of *M. tb* H37Rv, *M. bovis* AF2122/97 and BCG-Pasteur. (**A**) The upstream sequences of *phoP* from three strains are showed. Two SNPs are identified at the indicated positions (relative to the start codon) between *M. tb* and *M. bovis* or BCG-Pasteur. No sequence polymorphism is detected with the ORF of *phoP*. (**B**) Three SNPs between *M. tb* and *M. bovis* or BCG-Pasteur are identified at the indicated positions (relative to the start codon) within the ORO of *phoR*.

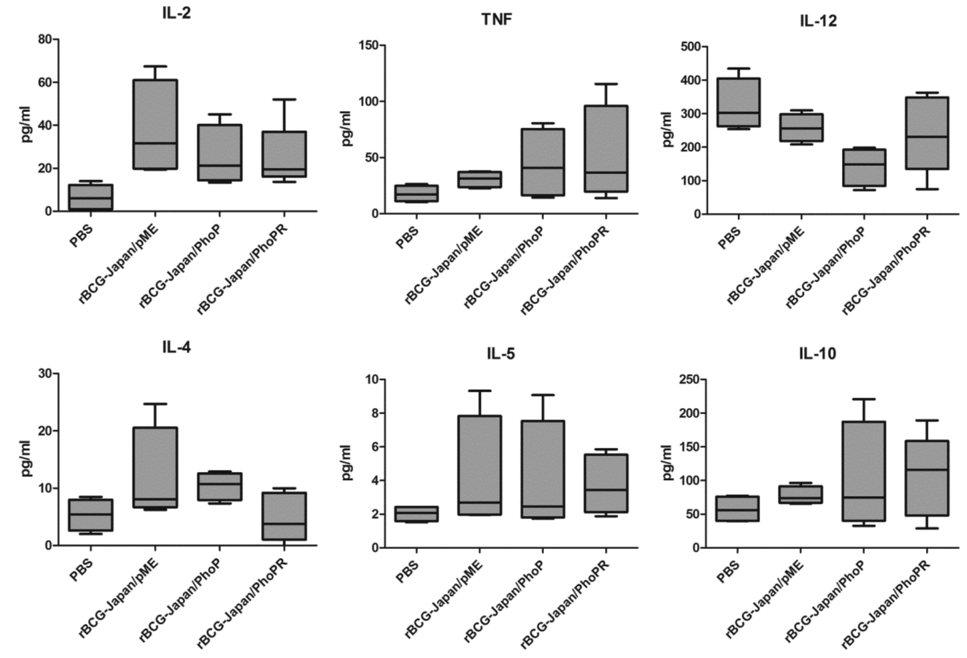

Figure S2. Immune response induced by recombinant BCG-Japan strains. C57BL/6 mice (n=4 per group) were immunized subcutaneously with 5×10^4 CFU of the indicated BCG strains. At 8 weeks post-vaccination, splenocytes were harvested and were incubated with or without PPD (10 μg/ml) for 72 hr and production of the indicated cytokines was analyzed by ELISA. Data are shown as mean ± SEM. No statistical difference was found among the four groups in each of the dataset.

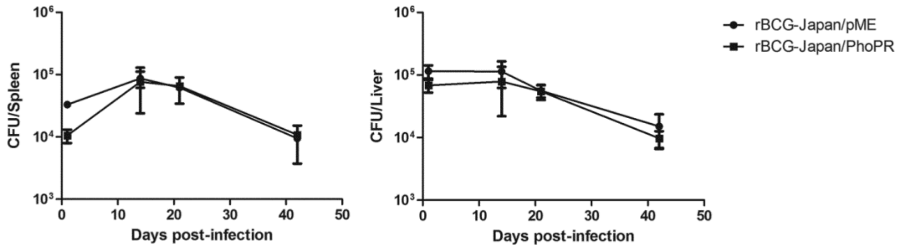

Figure S3. Replication of the recombinant BCG-Japan strains in immunocompetent mice. Groups of 4 male C57BL/6 mice were injected intravenously with 10^8 CFU of rBCG-Japan/pME or rBCG-Japan/PhoPR. At day 1, 14, 21, and 42 post-infection, mice were euthanized. The harvested organs were homogenized separately and plated on 7H11 agar to assess bacterial burden. Data are shown as mean ± S.D.

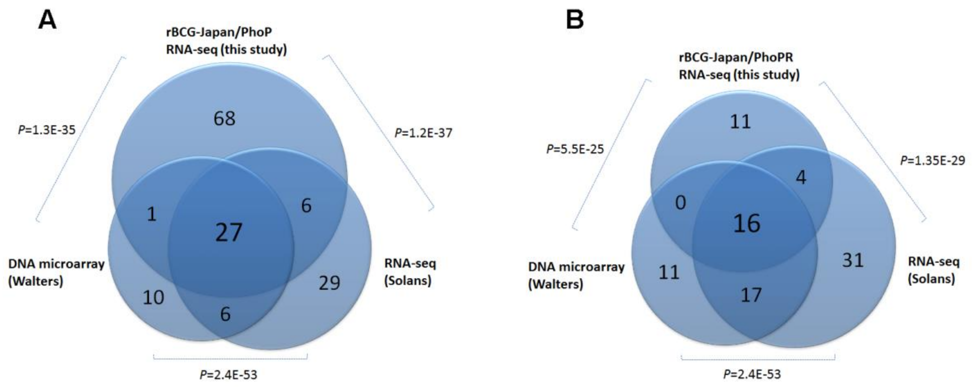

Figure S4. Overlap of PhoP activated genes identified from three separate studies.

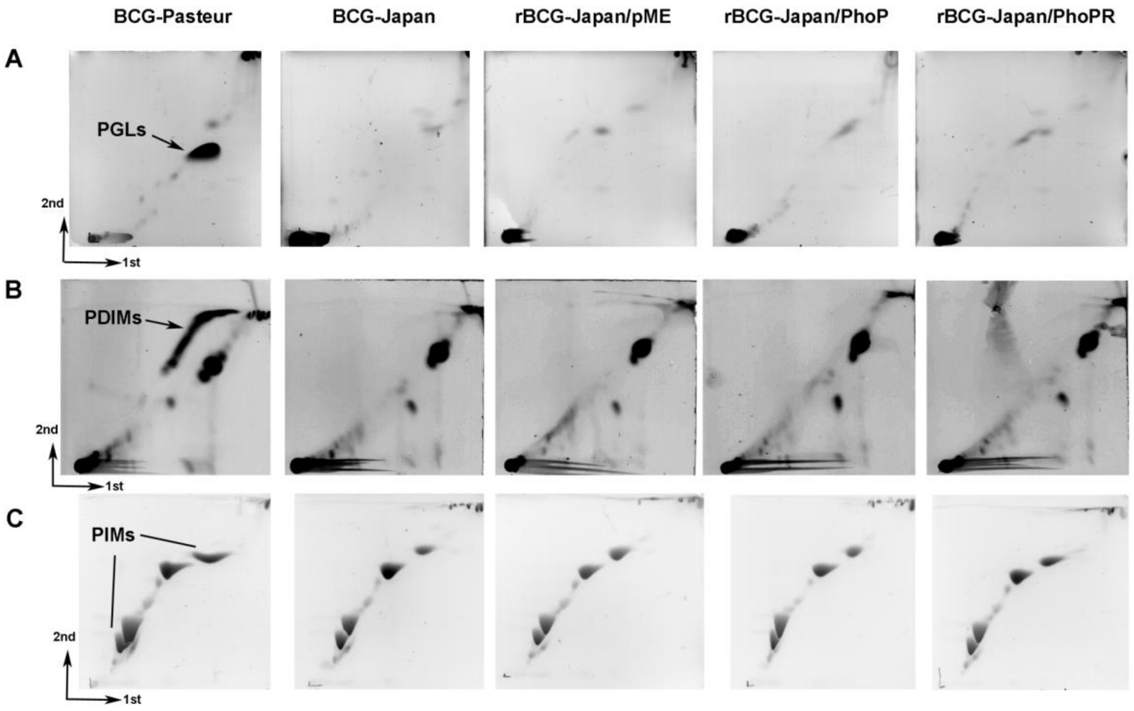

Figure S5. 2D-TLC analysis of cell wall lipids. (**A**) The apolar lipid extract was developed with the solvent system C: chloroform/methanol (96:4) in the first dimension and toluene/acetone (80:20) in the second dimension, followed by charring with α-naphthol. PGLs: phenolic glycolipids. (**B**) Apolar lipids were developed with the solvent system A: petroleum ether/ethyl acetate (98:2, v/v, 3×) in the first dimension and petroleum ether/acetone (98:2) in the second dimension, followed by charring with 5% phosphomolybdic acid. PDIMs: phthiocerol dimycocerosates. (**C**) Polar lipids were separated with the solvent system E: chloroform/methanol/water (60:30:6) in the first dimension and chloroform/acetic acid/methanol/water (40:25:3:6) in the second dimension, followed by charring with α-naphthol. PIMs: phosphatidylinositol mannosides.
